# Formulation and Characterization of Edible Bigel Inks for Structuring Fat Alternatives in 3D-Printed Foods

**DOI:** 10.3390/gels12030254

**Published:** 2026-03-18

**Authors:** Konstantina Zampouni, Theocharis Salamandrakis, Triantafyllia Biza, Thomas Moschakis, Eugenios Katsanidis

**Affiliations:** Department of Food Science and Technology, School of Agriculture, Faculty of Agriculture, Forestry and Natural Environment, Aristotle University of Thessaloniki, 54124 Thessaloniki, Greece; theocharisalamandrakis@gmail.com (T.S.); filio.mpiza@gmail.com (T.B.); tmoschak@agro.auth.gr (T.M.); ekatsani@agro.auth.gr (E.K.)

**Keywords:** agar, bigels, extrusion-based 3D printing, gelatin, hydrogel, oleogel, structured systems

## Abstract

Bigels (BGs) are promising biphasic systems for extrusion-based 3D food printing inks. In this study, BG inks were formulated by combining a 6% beeswax—4% monoglycerides oleogel (OG) with a 4% gelatin—1% guar gum hydrogel (HG). The BGs were formulated at OG:HG ratios of 10:90 up to 50:50. The effect of the OG:HG ratio on appearance, microstructure, extrusion, rheological and thermal characteristics was investigated to assess printability and shape fidelity. All formulations showed no signs of phase separation during storage, while changes in color were observed with increasing OG content, suggesting modifications in phase distribution and light-scattering behavior. Increasing the OG content induced a transition from OG-in-HG systems to a bicontinuous structure at a 50:50 ratio. All inks showed shear-thinning behavior (*G′* > *G*″) and viscoelastic properties suitable for 3D printing. BG with intermediate OG contents displayed moderate extrusion forces (7.27–9.00 N) and improved structural recovery (up to ≈60%), consistent with desirable printability and appropriate yield/flow points to ensure shape fidelity after deposition. Thermal analysis further confirmed the coexistence of OG and HG phases, ensuring structural integrity at printing temperature. These findings demonstrate the potential of BG as tunable, fat-reduced inks for 3D food structuring.

## 1. Introduction

Three-dimensional (3D) food printing is a digitally driven fabrication technology that enables the layer-by-layer deposition of food materials under precise computer control [1]. This approach enables the fabrication of highly customized and geometrically complex food products that are difficult to achieve using conventional processing techniques. By allowing precise control over shape, texture, appearance and nutritional composition, 3D food printing supports innovation in food design, personalized nutrition and improved sensory quality [2]. In addition, accurate portioning and on-demand fabrication offer potential sustainability benefits by minimizing food waste and reducing packaging requirements [3].

Various 3D printing techniques have been explored for food applications [4]; however, extrusion-based printing is the most widely used due to its simplicity, cost-effectiveness and compatibility with food materials [5]. Consequently, a large proportion of 3D food printing research has focused on extrusion-based techniques [6,7]. In this process, semi-solid formulations are extruded through a nozzle and deposited layer by layer to construct 3D structures [8]. Successful printing requires materials with suitable extrudability and sufficient mechanical strength to maintain their shape after deposition, making rheological and mechanical characterization essential. In particular, printable food inks should exhibit shear-thinning behavior to facilitate flow through the nozzle under applied stress, and rapidly recover their viscoelastic structure after deposition to ensure shape fidelity [9,10]. In addition, an appropriate balance between yield point and elastic modulus (*G′*) is required to prevent collapse or spreading of the printed layers, thereby ensuring dimensional accuracy and structural stability of the final construct [11]. Printing performance is further influenced by both material properties and processing parameters, which must be carefully controlled to achieve reproducible, high-quality printed structures [8,11].

Among the various food ingredients investigated for 3D printing applications, lipid-based systems, such as oleogels (OGs), have attracted attention due to their ability to substitute fats in foods [12,13]. Oleogelation is a structuring process in which liquid edible oils are transformed into semi-solid systems through the formation of a 3D network of oleogelators that immobilizes the oil phase without altering its chemical composition [14]. Beeswax (BW) and monoglycerides (MGs) are widely used food-grade oleogelators, valued for their crystallization ability and lipid-structuring properties in oil systems [15]. MGs, in particular, self-assemble into ordered crystalline structures, forming cohesive lipid networks with enhanced elasticity and mechanical strength [16,17]. When combined with BW, MGs exhibit synergistic structuring behavior, as their amphiphilic character promotes efficient crystal packing and improved network cohesion within the lipid phase [15].

Also, protein- [18] and polysaccharide-based hydrogels (HGs) have been extensively explored as edible inks for 3D food printing due to their biocompatibility, tunable rheological behavior and acceptance as food ingredients [19,20]. Gelatin (GEL), a natural protein derived from the partial hydrolysis of collagen [21], is particularly effective for 3D food printing applications because of its thermo-reversible gelation, favorable mouthfeel and consumer acceptance. GEL-based systems can form physically crosslinked networks through hydrogen bonding and triple-helix formation, providing structural integrity at ambient temperatures [22]. However, GEL HGs often exhibit limited mechanical strength under the high shear conditions of 3D printing, restricting their printability. To address this, polysaccharides are commonly incorporated to enhance viscosity, network stability and extrusion performance [23,24]. Guar gum (GG) is a cost-effective and widely used natural galactomannan polysaccharide in food applications. It is extracted from the endosperm of *Cyamopsis tetragonolobus* and consists of a β-(1→4)-linked D-mannopyranose backbone with α-(1→6)-linked D-galactopyranose side chains [25]. As a nonionic hydrocolloid with strong water-binding capacity, GG effectively thickens aqueous systems and enhances viscoelastic behavior through polymer entanglement and hydrogen bonding with water molecules. Previous studies have demonstrated that the addition of GG significantly improved the rheological properties and printing accuracy of other protein-based gels [26] and emulsion systems [27,28,29] used in 3D food printing.

Bigels (BGs) are biphasic gel systems composed of an OG and a HG phase within a single structured material [30]. By combining hydrophilic and lipophilic phases, BGs form a double-network system that allows modulation of mechanical, rheological and thermal properties critical for extrusion-based 3D printing. In food applications, BGs have been extensively studied due to their ability to simultaneously incorporate water-soluble and lipid-soluble components, including nutrients and bioactive compounds [31,32,33,34], and to function as fat replacers [35,36,37,38]. From a structural perspective, the HG phase provides a continuous, deformable matrix that facilitates the extrusion, while the OG phase acts as a strengthening agent that enhances elasticity, yield point and shape retention after deposition. Therefore, the resulting balance between flowability under shear and rapid structural recovery makes BG systems particularly appropriate materials for 3D printing applications [11,39,40]. However, despite the increasing research on BG systems, their design as fat-structured printable inks remains limited. In particular, the combined use of a BW–MGs OG within a GEL–GG hydrocolloid matrix and its systematic evaluation in relation to printability-related parameters has not been thoroughly investigated.

Therefore, the aim of this study was to develop and characterize edible BG-based inks that can be used for extrusion-based 3D food printing by combining a BW-MGs OG with a GEL-GG HG in different mixing ratios. Specifically, the effect of varying OG:HG ratios on the microstructure, thermal behavior, extrusion performance, rheological properties and stability of the resulting inks was investigated in order to evaluate their printability and the shape fidelity after deposition. This study would provide valuable information for the design and functional performance of edible BG inks as potential fat alternative inks for 3D printed food products.

## 2. Results and Discussion

### 2.1. Appearance and Stability of Inks

**Figure 1:**
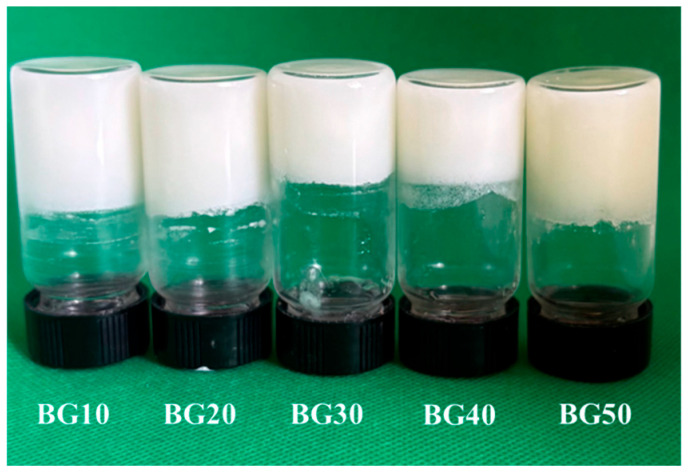
Visual appearance and physical stability of bigel (BG) ink formulations with different OG:HG ratios (10–50%), denoted as BG10–BG50, after storage under refrigerated conditions, were evaluated using the inverted tube method.

Visual evaluation indicated that the GEL–GG HG formed turbid systems with smooth and uniform surfaces, a behavior commonly associated with the incorporation of polysaccharides into GEL-based HG networks [22]. In contrast, the OG structured with BW and MGs appeared opaque and exhibited a slightly yellowish hue, reflecting the formation of a crystalline lipid network [17]. After the incorporation of the OG phase into the HG matrix under high-speed mixing, the resulting BG inks exhibited a macroscopically homogeneous appearance with a semi-solid consistency at 27 °C. The successful formation and physical integrity of the BGs were further confirmed using the inverted tube test at ambient temperature [41,42]. The absence of flow upon inversion confirmed the adequate self-supporting ability of all BG inks, with no signs of phase separation, which is essential for maintaining structural integrity and for their application as fat alternatives (Figure 1). These semi-solid BG inks are suitable for syringe-based 3D extrusion printing, as they can be smoothly extruded through the printing nozzles without difficulty. Moreover, visual observation of the BG inks over time confirmed that all formulations remained macroscopically homogeneous, with no visible signs of phase separation. Similar self-supporting BG formulations composed of BW and either GEL or agar in combination with xanthan gum have been previously reported in the literature [43].

Nevertheless, variations in color and surface morphology were associated with higher OG ratios, representative of the modifications in the internal structure of the binary system. As reported by Zampouni et al. [22], the color of a BG system is mainly influenced by light diffraction from oil droplets, the dispersed phase size distribution and the OG:HG ratio, while the scattering behavior is closely related to the concentration of the internal phase and the size of the oil droplets.

**Figure 2:**
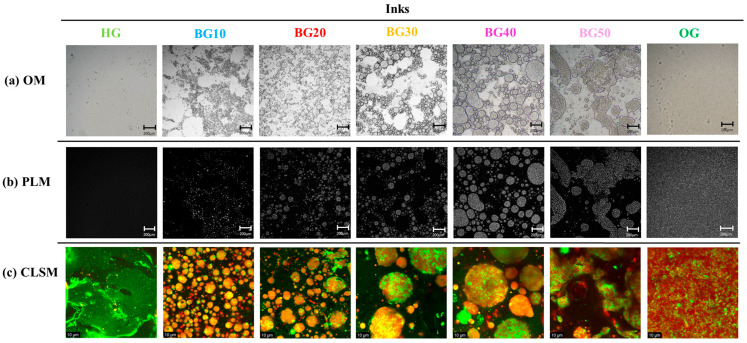
Microstructure of hydrogel (HG), oleogel (OG) and bigel (BG) ink formulations with different OG:HG ratios (10–50%), denoted as BG10–BG50, observed using (**a**) optical microscopy (OM, scale bar: 200 μm), (**b**) polarized light microscopy (PLM, scale bar: 200 μm) and (**c**) confocal laser scanning microscopy (CLSM, scale bar: 10 μm).

The results of the instrumental color attributes for the HG, OG and BG formulations are summarized in Table 1. The OG:HG ratio affected the lightness (L*) values of the different formulations. BG containing low OG fractions, namely BG10 and BG20, showed the highest L* and *WI* values, indicating bright and whiter structures. This behavior is consistent with the microstructural observations discussed in detail in the following section, where finely dispersed OG droplets were observed within a continuous HG matrix that enhance light scattering. As the OG content increased, a gradual decrease in L* and *WI* values was observed for BG30 and BG40. A significant reduction in L* values was evident for BG50 (59.02 ± 1.01), approaching values similar to the OG (61.49 ± 3.05). This behavior may reflect a transition from finely dispersed OG droplets within a continuous HG matrix to systems characterized by larger lipid domains, suggesting a bicontinuous structure (Figure 2). All BG formulations exhibited negative a* values, indicating a slight shift toward green hues. The degree of the negative a* parameter increased with increasing OG content, from −0.32 ± 0.03 in the HG to −3.27 ± 0.24 in the OG.

The a* values of the BG inks became gradually more negative from BG10 (−0.98 ± 0.04) to BG50 (−2.94 ± 0.26), suggesting an increasing contribution of the lipid phase to green color development. Minor deviations were observed between intermediate formulations (e.g., BG20 and BG30), but they did not affect the overall trend. Similar findings have been reported for BG systems structured with GG and rice bran wax, using walnut oil as the OG phase [44]. The b* parameter, representing yellowness, showed formulation-dependent variations. Among the BG inks, BG40 exhibited the highest b* (7.61 ± 0.96) and C* values, indicating enhanced color saturation, whereas BG50 showed lower b* (3.91 ± 1.15) and C* values. This reduction is attributed to the heterogeneous phase distribution occurring at near-equal OG to HG ratios, which limits efficient light scattering in the yellow wavelength region.

### 2.2. Microstructure of Inks

The microstructure of the ink formulations designed for 3D food printing was investigated by optical microscopy (OM), polarized light microscopy (PLM) and confocal laser scanning microscopy (CLSM) to elucidate the effect of the OG:HG ratio on phase organization and dispersion of BG inks. The HG, composed solely of GEL, GG and water, exhibited a homogeneous and continuous aqueous network without discernible dispersed domains, confirming the formation of a single-phase system (Figure 2). In contrast, the OG, consisting of sunflower oil structured with BW and MGs, displayed a dense and uniform microstructure characterized by finely distributed birefringent crystalline entities, indicative of the formation of a continuous lipid crystalline network [15].

BG ink formulations containing up to 40% OG (BG10–BG40) exhibited a clearly biphasic microstructure, in which the OG phase was dispersed as oil droplets within the continuous HG matrix. Similar BG systems with OG-in-HG organization for 3D printing applications have been reported in the literature [45,46]. Also, OM images (Figure 2a) revealed an increase in oil droplet size with increasing OG fraction, resulting in significant differences in color parameters (Table 1). At low OG ratios (BG10 and BG20), where the HG phase predominated, small, nearly spherical OG droplets were uniformly distributed, indicating an efficient homogenization process [22]. This behavior can be attributed to the higher relative concentrations of GEL and GG, which enhanced the viscosity and stabilizing capacity of the continuous phase. As the OG content increased to 30% (BG30), the droplets became significantly larger and more closely packed, suggesting increased phase interactions, while still maintaining a discrete droplet morphology. At higher OG levels (BG40), droplet enlargement became more evident, suggesting the lower availability of hydrocolloids to stabilize the dispersed lipid phase. When the OG:HG ratio reached 50:50 (BG50), OM images revealed the formation of irregularly shaped domains, characteristic of a bicontinuous-type BG structure [47,48]. The term bicontinuous has been previously used to describe the structural arrangement in which both OG and HG phases form interpenetrating, continuous networks within the BG system [17]. This transition is indicative of the onset of oil droplet coalescence phenomena in BG50, leading to a more interconnected internal network in which neither phase is clearly continuous within the system [49].

Additionally, PLM enabled a more comprehensive understanding of the internal organization of the OG phase of BG inks (Figure 2b). The presence of birefringent regions in all BG formulations confirmed the formation of composite crystalline BW and MGs structures within the dispersed OG domains, similar to the plain OG system [17]. Moreover, both the intensity and spatial distribution of birefringent areas increased with increasing OG content, indicating a progressively stronger contribution of the lipid crystalline network to the overall microstructure. This observation confirmed that the crystalline integrity of the OG phase was well-maintained after the BG formation and that the lipid phase was effectively incorporated within the hydrocolloid matrix of GEL and GG, which is critical for the development of structured food systems with tailored textural properties.

CLSM images (Figure 2c) further elucidated the spatial distribution of the two phases, with the HG appearing as a continuous green matrix and the OG phase visualized as red/orange domains. At low OG concentrations (BG10–BG20), CLSM images confirmed a fine and homogeneous dispersion of small oil droplets within the HG network. Similar observations have been reported for BG systems composed of κ-carrageenan and glycerol monostearate with BW, where intermediate formulations exhibited larger oil droplets in proximity, indicating enhanced interactions [50]. Consistent with these findings, the intermediate ink formulations in the present study (such as BG30 and BG40) showed larger oil droplets, suggesting improved structuring between the OG and HG phases. In contrast, previous studies on OG-in-HG BG have reported variations in droplet size with increasing concentrations of candelilla wax in the OG phase [51]. At higher OG contents, BG50 exhibited significantly enlarged OG regions with irregular boundaries and partial coalescence, indicating reduced dispersion stability and supporting a transition towards bicontinuous morphology. Similarly, Qiu et al. [52] observed a semi-bicontinuous morphology at equal OG and HG proportions, while increasing the OG content to 70% resulted in an HG-in-OG structure due to progressive phase inversion.

### 2.3. Forward Extrusion Properties of Inks

The extrusion force parameters constitute an initial evaluation of the printing ability of inks at 27 °C and their behavior during extrusion-based 3D food printing [53]. According to Table 2, significant differences were observed for peak extrusion force, extrusion force at fixed displacements, work of extrusion and firmness among the different inks (*p* < 0.05). These differences can be attributed to the microstructural variations identified among the ink formulations. Specifically, changes in lipid domain size, phase distribution and the degree of structural continuity between the OG and HG phases influence the internal resistance of the system to deformation. Formulations characterized by larger (BG40) or more interconnected lipid domains (BG50) exhibited increased resistance to flow, resulting in higher extrusion forces, whereas BG systems with finely dispersed lipid droplets (BG10 and BG20) showed lower resistance and improved flowability during extrusion. In more detail, BG10 exhibited the lowest peak extrusion force as well as the lowest force values at 5 and 10 mm, indicating that this formulation flows readily under applied stress. Such behavior is useful for reducing energy consumption and mechanical load on the printing system. However, excessively low extrusion forces may also reflect limited structural resistance, which can compromise shape fidelity after deposition. In contrast, BG50 required the highest extrusion forces across all examined parameters, reflecting a highly resistant structure that demands increased pressure to initiate and sustain flow. While this can improve filament stability, it may negatively affect printing efficiency by increasing the risk of nozzle clogging and discontinuous extrusion. According to Perera et al. [54], the harder and stronger gels are more challenging to extrude in 3D printing. HG showed relatively high extrusion forces, comparable to BG40 and OG, indicating a strong resistance to flow initiation. This suggests that HG possesses a cohesive network that supports dimensional stability but may require higher pressures during the printing process. BG20 and BG30 exhibited intermediate behavior, with moderate extrusion forces that balance ease of flow and structural robustness, pointing to more favorable and desirable processing characteristics.

The work required for extrusion followed a similar trend to that of extrusion force, with BG10 displaying the lowest values and BG50 the highest. Work represents the total mechanical energy needed to extrude the material and is therefore a critical indicator of process efficiency. The low work values of BG10 and BG20 suggest efficient extrusion with minimal energy input, whereas the high work values of BG50 indicate a more energy-intensive process. Additionally, firmness values reflect the resistance of the extruded material to deformation, which is directly linked to shape retention. BG10 exhibited the lowest firmness, consistent with its low extrusion force and weak resistance to deformation, suggesting a tendency toward filament spreading and poor edge definition after deposition. In contrast, BG50 showed the highest firmness, indicating strong resistance to deformation and the ability to maintain printed geometries. The HG, BG30, BG40, and OG inks exhibited sufficient firmness to maintain shape fidelity without excessive rigidity. In contrast, BG10, despite its excellent extrudability, showed inadequate mechanical stability for high-resolution printing. BG50 demonstrated superior firmness and filament stability; however, it required substantially higher extrusion pressures, which may hinder continuous printing and increase mechanical stress on the printing system. Among the tested formulations, BG20 and BG30 appeared to be the most efficient, as they combined moderate extrusion forces with adequate firmness and work values. This balance is critical for extrusion-based 3D food printing, where both smooth material flow through the nozzle and reliable shape retention after deposition are essential.

### 2.4. Rheological Behavior of Inks

#### 2.4.1. Flow Behavior and Apparent Viscosity Profiles

**Figure 3:**
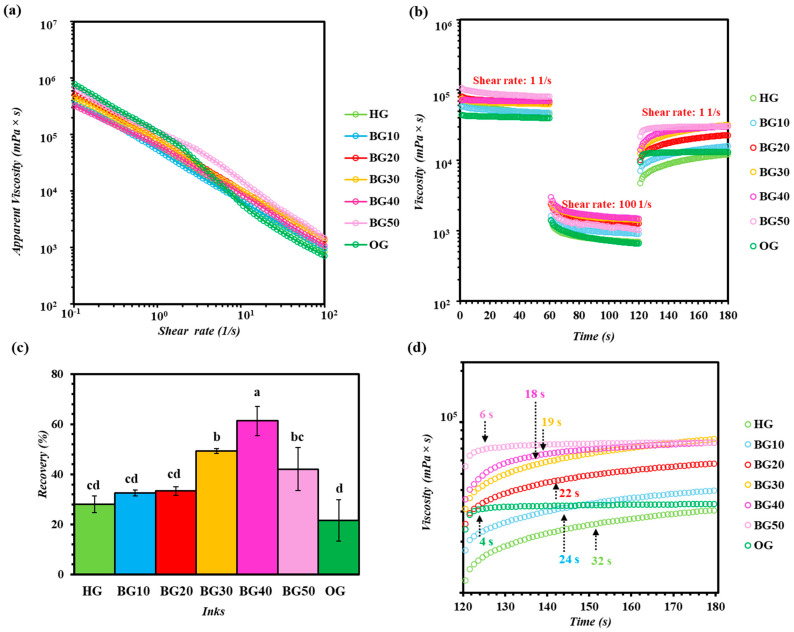
Rheological behavior of hydrogel (HG), oleogel (OG) and bigel (BG) ink formulations with different OG:HG ratios (10–50%), denoted as BG10–BG50: (**a**) viscosity versus shear rate, (**b**) three-interval thixotropy test (shear rate: 1–100–1 1/s), (**c**) recovery percentage and (**d**) viscosity recovery time at the third interval. Values are presented as mean ± SD. Different letters indicate significant differences in recovery (%) among inks (*p* < 0.05).

The flow behavior of the HG, OG and BG ink formulations was evaluated by steady shear measurements over a shear rate range of 1–100 1/s and the corresponding viscosity profiles are presented in Figure 3a. All BG inks exhibited shear-thinning behavior, characterized by a continuous decrease in viscosity with increasing shear rate [55]. As shown in Figure 3a, the apparent viscosity declined sharply as the shear rate increased, indicating that all formulations behaved as non-Newtonian fluids with strong pseudoplastic characteristics. This behavior is significant for extrusion-based 3D printing, as it facilitates material flow through the nozzle under high shear while enabling shape retention once shear is removed [11,56].

The plain HG and OG displayed distinct rheological responses, reflecting their different structural organizations. Specifically, HG exhibited a predominantly polymeric network–dominated behavior, whereas OG viscosity was regulated by the formed crystalline lipid network. Regarding BG ink formulations, at low shear rates, viscosity increased progressively with increasing OG content, particularly for BG30 and BG40, indicating enhanced resistance to flow. This increase can be attributed to the microstructural development observed in these ink formulations, where larger and more closely packed OG droplets are embedded within the HG matrix as confirmed by microstructural analysis (Figure 2), leading to stronger interfacial interactions. At higher shear rates, all ink formulations showed a significant reduction in viscosity, indicating efficient structural breakdown under applied shear. This response implies the deformation of the HG network together with the disruption and rearrangement of the dispersed OG domains. Notably, formulations with intermediate OG contents (BG30 and BG40) maintained low viscosities at high shear rates, suggesting favorable extrusion behavior without excessive resistance during the 3D printing process. In contrast, BG10 and BG20, characterized by finely dispersed and smaller OG droplets within a dominant HG phase (Figure 2), exhibited lower viscosities across the shear rate range. While this behavior favors easy flow, it may limit post-extrusion shape stability due to reduced structural support from the OG phase. On the other hand, BG50 showed relatively high viscosity at low shear rates but a weaker shear-thinning response, which can be associated with its heterogeneous and partially bicontinuous microstructure (Figure 2). Such structural heterogeneity may hinder uniform stress distribution and result in erratic flow behavior during extrusion.

The Ostwald-de Waele power law parameters (Table 3) indicated significant differences in flow behavior among the formulations (*p* < 0.05). HG and BG10–BG30 inks exhibited moderate consistency indices (*K*) and low flow behavior indices (*n* ≈ 0.16–0.17), showing significant shear-thinning behavior, typical of hydrocolloid-based systems. The observed shear-thinning behavior can be explained by the alignment and disentanglement of polymer chains (GEL and GG), accompanied by the reversible disruption of physical junctions under applied shear stress. As the OG fraction increased (BG40–BG50), *K* values increased significantly, reflecting higher resistance to flow due to reduced HG content and the increased contribution of the OG phase. The significant decrease in *n* values for BG50 ink indicated increased shear-thinning, consistent with the rapid breakdown of a dense but physically cross-linked network during deformation. In contrast, OG exhibited the highest *K* values and negative *n* values, suggesting an undesirable flow behavior associated with the observed structural instability. This indicated that, although OG forms a mechanically rigid system, its flow response deviates from classical Ostwald-de Waele power law behavior, limiting the predictive capability of this model for purely lipid-structured systems.

#### 2.4.2. Thixotropic Behavior and Structural Recovery

The thixotropic behavior and structural recovery of the HG, OG and BG ink formulations were evaluated via a three-interval thixotropy test (3ITT), simulating the shear conditions during extrusion-based 3D food printing (Figure 3b,c). The 3ITT protocol includes an initial low shear interval (1 1/s) representing the material at rest, a high shear interval (100 1/s) simulating the extrusion process through the nozzle and a final low shear interval (1 1/s) to assess post-shear structural recovery [57]. During the first low-shear interval, all formulations exhibited relatively high viscosity values, reflecting the integrity of their internal network structures. Upon application of high shear (100 1/s), a rapid decrease in viscosity was observed for all inks, indicative of the structural breakdown and confirming their shear-sensitive behavior. This decrease in viscosity is essential for facilitating smooth extrusion during 3D printing. Following the removal of high shear, all inks exhibited partial viscosity recovery during the final low-shear interval, demonstrating their ability to rebuild their internal structure. However, the extent of recovery was significantly dependent on formulation composition (Figure 3d). BG formulations with intermediate OG contents exhibited significantly higher recovery compared to both the OG and HG. In more detail, BG30 and BG40 showed the highest recovery values, with BG40 exhibiting the maximum recovery (≈60%), indicating rapid and efficient network reformation after shear end. This improved recovery can be attributed to the synergistic interaction between the continuous HG matrix and the dispersed OG droplets, which act as reinforcing elements and promote elastic rebuilding. In contrast, BG10 and BG20 showed lower recovery values (≈30–35%), likely due to the dominance of the HG phase and the limited impact of the OG network on post-shear structural reconstruction. Although BG50 exhibited higher recovery values than BG10 and BG20, its heterogeneous and bicontinuous microstructure may not be desirable for extrusion-based 3D food printing, as it can hinder uniform stress redistribution and compromise flow homogeneity despite the improved recovery behavior. The lowest recovery was observed for OG, suggesting reduced structural resilience [58]. However, contrasting results have been reported for recovery behavior in different BG systems, with the recovery rate decreasing markedly as the OG:HG ratio increased [59]. This indicates that the recovery behavior of BG inks is influenced not only by the OG:HG ratio, but also by the type and concentration of the structuring agents used in each structured system.

According to Liu et al. [60] and Tian et al. [61], the recovery time of printable inks is a significant parameter for extrusion-based 3D printing, as a shorter recovery time indicates faster reformulation of the structure and better mechanical properties, which are essential for supporting subsequently deposited layers and maintaining shape fidelity. The recovery time of the BG inks after high shear deformation was evaluated during the final low-shear interval (1 1/s) of the 3ITT protocol (Figure 3d). In particular, recovery time was defined as the time required for the viscosity to reach a plateau value following the removal of high shear [57]. Significant differences in recovery time were observed among the ink formulations. BG50 showed the shortest recovery time (~6 s), followed by BG40 (~18 s) and BG30 (~19 s), indicating rapid reformation of the internal network after shear removal. The fast recovery suggests efficient re-establishment of interfacial interactions between the dispersed oil droplets and the continuous HG matrix, in agreement with literature reports describing synergistic phase interactions in BG systems [60,61]. The stable viscosity observed after extrusion suggests adequate self-supporting ability of the BG inks [57]. In contrast, BG10 and BG20 required longer times to recover their viscosity (~24 s and ~22 s, respectively). This behavior can be attributed to the dominance of the HG phase, where strong shear disrupts the polymeric network and the reformation of hydrogen bonds requires additional time. The HG exhibited the slowest recovery (~32 s), reflecting the intrinsically time-dependent reorganization of the GEL–GG network after deformation, suggesting partial collapse of the printed structure and limited structural recovery. The OG showed a rapid apparent response at low viscosity levels. However, its overall recovery remained limited, consistent with its low recovery percentage and the absence of a continuous polymeric network capable of elastic rebuilding. BG50 displayed intermediate recovery times but a reduced final recovery level, indicating that although partial viscosity rebuilding occurs relatively quickly, the heterogeneous and partially bicontinuous microstructure restricts complete network regeneration.

#### 2.4.3. Strain-Dependent Viscoelastic Properties

**Figure 4:**
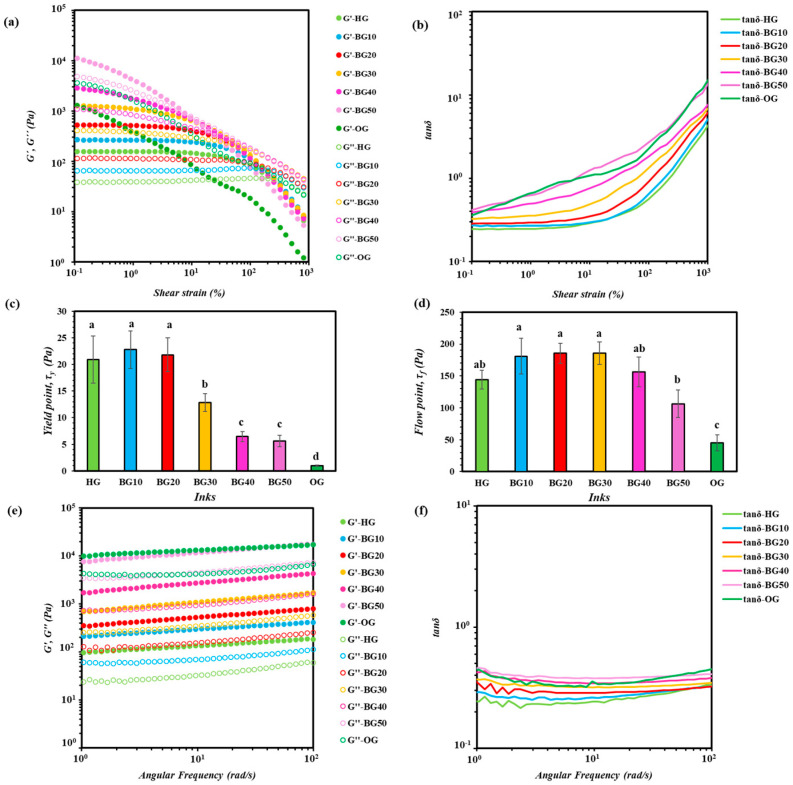
Rheological behavior of hydrogel (HG), oleogel (OG) and bigel (BG) ink formulations with different OG:HG ratios (10–50%), denoted as BG10–BG50: (**a**) storage modulus (*G*′) and loss modulus (*G*″) as a function of shear strain (%), (**b**) loss tangent (*tanδ*) as a function of shear strain (%), (**c**) yield point (*τ_ᵧ_*), (**d**) flow point (*τ_f_*), (**e**) storage (*G*′) and loss (*G*″) moduli as a function of angular frequency (rad/s) and (**f**) loss tangent (*tanδ)* as a function of angular frequency (rad/s). Values with different superscript letters for measured yield point and flow point are significantly different among ink formulations (*p* < 0.05).

At low shear strains, all inks exhibited *G′* > *G″* and low *tanδ* values (Figure 4a,b), confirming an elastic-dominated, gel-like behavior. HG exhibited the largest linear viscoelastic region (LVR), indicating higher structural stability. In contrast, all BG formulations showed lower LVRs than HG, demonstrating that the incorporation of OG into the HG matrix decreased resistance to deformation. Specifically, increasing the OG:HG ratio led to a progressive reduction in LVR, indicating increased shear sensitivity. OG exhibited the lowest LVR, reflecting its limited structural stability. Similar results have been reported for carboxymethyl-cellulose-ethyl-cellulose BG systems [54].

This indicates that the polymeric components form a well-developed network capable of storing mechanical energy. The higher initial *G*′ values observed for BG40 and BG50 suggest the formation of a denser and more strongly interconnected structure, due to enhanced intermolecular interactions such as hydrogen bonding, chain entanglement and physical crosslinking. These findings are in agreement with previous studies reporting that BGs composed of structured OG dispersed within HG matrices exhibit enhanced elastic moduli, attributed to the reinforcing role of the solid lipid network [52,62]. As strain increased, a progressive decrease in *G*′ values together with a concomitant increase in *tanδ* reflected the gradual disruption of this network. The strain level at which *tanδ* approached or exceeded unity exhibits the transition from elastic- to viscous-dominated behavior, corresponding to the onset of yielding. OG reached this transition at much lower strains, indicating a weak and easily disrupted structure. From a printing perspective, this behavior is advantageous, as materials that preserve elasticity under deformation are better able to retain their shape immediately after extrusion.

Yield point (*τ_ᵧ_*) plays a significant role in influencing the beginning (initiation of flow) and end (ink cut-off) of the printing process [61]. According to Figure 4c, the HG, BG10 and BG20 inks exhibited the highest *τ_ᵧ_* values, indicating strong resistance to flow initiation, which contributes to reducing filament collapse and improving shape retention. Nevertheless, excessively high *τ_y_* values may require higher force and lead to delayed or discontinuous material flow. BG30 displayed an intermediate response, offering a favorable compromise between resistance to flow at rest and ease of extrusion. In contrast, BG40 and BG50 showed lower *τ_y_* values, enabling smoother flow initiation but possibly reducing resistance to deformation immediately after deposition. OG, having the lowest *τ_y_* values, lacked sufficient structural integrity to ensure dimensional control, making it unsuitable for precision printing applications. Additionally, the flow point (*τ_f_*) of the ink formulations was evaluated to assess their self-supporting ability and shape fidelity during and after printing [54]. The *τ_f_* indicates the resistance of the inks to structural collapse under applied stress (Figure 4d) and is therefore directly related to printability and structural stability. BG10–BG30 exhibited the highest *τ_f_* values, indicating superior resistance to deformation and an enhanced capacity to support successive layers without collapse. BG40 and BG50 showed moderate *τ_f_* values, suggesting adequate but reduced load-bearing capacity, whereas OG displayed the lowest *τ_f_*, consistent with poor stacking stability. Therefore, the moderate OG incorporation in HG improves layer stacking by balancing flowability and mechanical strength, while excessive OG content compromises the ability to construct tall, self-supporting structures.

### 2.5. Frequency-Dependent Viscoelastic Properties

The frequency sweep results (Figure 4e,f) revealed that all inks maintained elastic dominance over the tested frequency range, confirming their ability to preserve filament integrity across the inferred angular frequency range [63]. Both moduli increased slightly with frequency, suggesting frequency-dependent reinforcement of the internal network structure [64]. BG40 and BG50 exhibited the highest *G*′, indicative of increased network rigidity. This observation was consistent with the higher extrusion forces recorded for these formulations in the forward extrusion test (Table 2), indicating greater resistance to deformation under applied stress. In contrast, HG, BG10 and BG20 showed lower *G*′ values, which corresponded to reduced extrusion forces and enhanced flowability. Additionally, the Weak gel model adequately described the viscoelastic structure of all ink formulations (*R*^2^ values reported in Table 3), revealing the development of network strength and structural organization depending on the OG:HG ratio [52]. The structural parameter *A* reflects the overall strength of the gel network and is associated with the density and intensity of intermolecular interactions within the BG system. The low *A* values observed for HG and BG10 indicated a weak network due to the presence of reversible polymer–polymer interactions and hydrogen bonding. As the OG content increased (BG20–BG40), *A* values increased, suggesting a strengthening of the gel structure due to the combined effects of BW and MGs and the development of lipid-associated structural areas. These higher *A* values were associated with increased mechanical resistance, in agreement with the elevated extrusion forces observed. BG50 showed a further increase in *A* values, with improved resistance to deformation but reduced cohesive strength against flow initiation. However, the high extrusion force and reduced homogeneity of BG50 suggest that network strengthening may compromise printability. The interaction exponent *z* provides information on the degree of network connectivity and the sensitivity of the structure to deformation. The OG ink exhibited the highest *A* and *z* values, indicating a different gel organization due to the presence of lipid crystal networks of BW and MGs and strong interactions between the oleogelators [52,57]. According to Qiu et al. [52], higher *A* values usually indicate stronger interactions between the OG and HG phases, arising from partial interpenetration of the two networks. This structural integration is associated with increased viscoelastic strength, higher yield stress and improved mechanical stability, which favor layer-by-layer deposition during extrusion-based printing. Also, the high *z* values suggest a higher sensitivity of the network to deformation, consistent with a rigid but less cohesive structure.

### 2.6. Thermal Behavior of Inks

**Figure 5:**
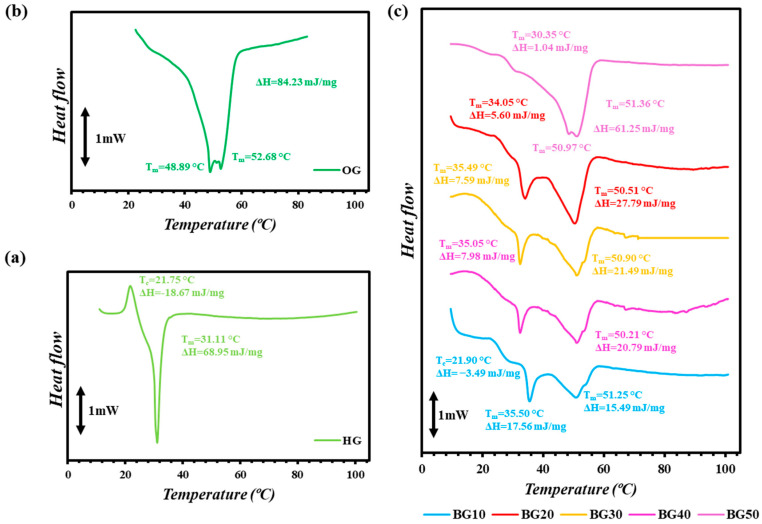
Representative thermograms of (**a**) hydrogel (HG), (**b**) oleogel (OG) and (**c**) bigel (BG) ink formulations with different OG:HG ratios (10–50%), denoted as BG10–BG50. Melting (*T_m_*) and crystallization (*T_c_*) temperatures with their corresponding enthalpy changes (Δ*H*) are indicated on the curves.

The thermal behavior of the OG, HG and BG ink formulations was investigated, and the corresponding thermograms are presented in Figure 5. Distinct thermal transitions were observed for each phase, reflecting their different structural organizations, whereas the resulting BG systems exhibited combined and composition-dependent thermal behavior. Compared to HG and OG, the BG formulations displayed more complex thermal profiles, with melting processes distributed over a broader temperature range [65].

The HG inks exhibited two characteristic thermal transitions. The first endothermic peak at around 21–22 °C can be attributed to the disruption of weak physical associations and partial rearrangement within the GEL-GG HG network, while the second, more intense peak at approximately 31 °C corresponds to the melting of GEL triple-helix junction zones (Figure 5a) [22]. The presence of GG did not establish distinct melting transitions but contributed to the stabilization of the HG network through polymer entanglement and water hydrogen bonding, as reflected in the relatively high enthalpy of the main transition. Additionally, the OG inks displayed two dominant endothermic melting peaks at approximately 48.89 °C and 52.68 °C, respectively, associated with the melting of the crystalline lipid network formed by the BW and MGs (Figure 5b). The relatively high cumulative ΔH indicated the formation of a well-organized and thermally stable crystalline network, consistent with the strong oil immobilization and solid-like behavior of OG. A similar observation was reported by Dimakopoulou et al. [66] for sunflower oil OGs structured with sunflower wax and MGs.

In contrast, the BG inks (BG10–BG50) exhibited dual thermal transitions, indicating the coexistence of the HG and OG networks within the same system. A low-temperature endothermic peak, typically observed between 21 and 36 °C, was associated with the thermal disruption of the HG phase, while a second peak at higher temperatures (approximately 50–52 °C) corresponded to the melting of the lipid crystalline network. The relative intensity and enthalpy of these transitions were strongly dependent on the OG:HG ratio. As the OG content increased, the ΔH associated with the high-temperature lipid melting peak increased, indicating a progressively greater contribution of the BW-MGs crystalline network to the structure of the BG systems. Conversely, the ΔH of HG-related transition decreased, reflecting the partial disruption of the continuous HG matrix at higher OG fractions. This trend was consistent with the microstructural observations (Figure 2), where increasing OG content led to larger oil droplets and a transition toward bicontinuous morphology at high ratios (BG50). Similarly, two distinct endothermic peaks were observed by [22,67], corresponding to the melting of the GEL HG phase and the lipid crystalline phase, respectively.

All BG formulations exhibited semi-solid-like characteristics at 27 °C, the temperature at which rheological and extrusion measurements were performed. At this temperature, the GEL-GG HG networks remained partially structured, while the lipid crystalline network was fully solid. This thermal state explains the favorable balance between flowability under shear and structural recovery observed in the rheological tests.

## 3. Conclusions

The present study demonstrated that the controlled incorporation of a BW–MGs OG into a GEL–GG HG matrix enables the development of structurally stable BG-based inks suitable for extrusion-based 3D food printing. The OG:OH ratio was identified as a critical formulation parameter, significantly influencing extrusion behavior, viscoelastic recovery and post-deposition shape fidelity. Among the investigated systems, intermediate formulations (BG30–BG40) achieved the most favorable balance between flowability and mechanical integrity, ensuring consistent extrusion performance and structural stability. In contrast, low-OG formulations exhibited limited resistance to deformation, whereas high-OG systems required high extrusion forces and showed reduced structural homogeneity. These findings highlight the importance of controlled multiphase structuring through lipid–hydrocolloid interactions as a formulation strategy for designing tunable, fat-structured edible inks for advanced food manufacturing applications. Although this study was performed under controlled laboratory-scale conditions in order to evaluate the physicochemical and rheological characteristics, additional validation through full-scale 3D printing trials and product-level performance evaluation would further support the practical applicability of the proposed systems.

## 4. Materials and Methods

### 4.1. Oleogel Preparation

For the preparation of OG, sunflower oil (Minerva SA, Metamorphosis, Greece) was used as the lipid phase. Beeswax (BW, white pharma grade, Syndesmos S.A., Athens, Greece) and monoglycerides (MGs, HARI 95 distilled monoglycerides, Rikevita SDN BHD, Johor Bahru, Malaysia) were utilized as oleogelators to structure the sunflower oil. The MGs were derived from edible, fully hydrogenated palm oil and were commercially available as a white powder with a purity of 95%. Initially, the sunflower oil was weighed into a beaker and heated to 85–90 °C under continuous stirring using a magnetic stirrer (3 Pro) (LGG Labware Co., Ltd., Beijing, China). Subsequently, 6% *w*/*w* BW and 4% *w*/*w* MGs were weighed and gradually added to the sunflower oil at 85 °C to avoid a significant temperature drop. After the addition of the oleogelators, the samples were maintained at 90 °C for 30 min to ensure complete dissolution and homogenization [17,68]. For separate characterization, the molten OG was transferred into plastic containers and allowed to cool at ambient temperature for 45 min, followed by storage at 4 °C for 24 h. Prior to analysis, the OG was equilibrated at 27 °C for 2 h in a temperature-controlled chamber.

### 4.2. Hydrogel Preparation

For the preparation of HG, distilled water was used as the aqueous phase, while 4% *w*/*w* gelatin (GEL, pork gelatin, type A, 270 Bloom, RAPS GmbH & Co., KG, Kulmbach, Germany) and 1% *w*/*w* guar gum (GG, AG 20/50, 200 mesh, 5000–5500 cps, food grade; Agro Gums, Ahmedabad, India) were employed as hydrogelators. The beaker containing distilled water was placed on a magnetic stirrer under continuous agitation and was gradually heated. The hydrogelators were slowly added at 40 °C to avoid a significant temperature decrease. The mixture was then further heated to 80 °C and maintained under continuous stirring at this temperature for at least 10 min to ensure complete dissolution of both hydrogelators [17,22]. For the analysis and characterization of the plain HG, samples were cooled in an ice bath for 45 min, followed by storage at 4 °C for 24 h. Before further analysis, the HG was equilibrated at 27 °C for 2 h in a temperature-controlled chamber.

### 4.3. Bigel Preparation

BG inks were prepared by combining OG and HG phases at different ratios (10:90-50:50), as summarized in Table 4. Initially, the OG and HG were prepared separately following the procedures described above. For BG formation, the molten OG (80 °C) was gradually added to the HG phase (75 °C) under controlled conditions. The addition was performed at a slow rate to promote uniform dispersion, followed by high-shear homogenization using an Ultra-Turrax homogenizer (IKA, Staufen, Germany) at 10.000 rpm for 2 min. Throughout the homogenization process, the system temperature was carefully maintained at 75 °C to ensure both phases remained in the molten state and to facilitate effective incorporation of the OG into the HG matrix. After the homogenization, the resulting BG inks were immediately transferred into plastic containers and cooled in an ice bath for 45 min to induce gelation. The samples were then stored at 4 °C for 24 h to allow for full structural organization and were subsequently equilibrated at 27 °C for 2 h in a temperature-controlled chamber before further characterization. BG treatments with OG contents above 50% were not further investigated, as preliminary experiments indicated phase separation, which prevented the formation of self-standing BG inks for 3D printing.

### 4.4. Stability and Color Evaluation

Aliquots of the homogenized hot inks were transferred into glass vials, immediately cooled in an ice bath for 45 min, and subsequently stored under refrigerated conditions (5 °C) for up to 60 days to assess long-term structural stability and visual appearance. The stability of the different ink formulations was periodically evaluated during refrigerated storage (5 °C) using the inverted tube test [30,69]. Briefly, samples were stored in sealed glass vials and were inverted at predetermined time intervals to assess structural integrity. The absence of flow, phase separation or visible structural collapse upon inversion was considered indicative of physical stability. In addition, samples were visually examined for any signs of microbial or mold growth throughout the storage period.

Color characteristics of the inks were measured using a Chroma CR-400 colorimeter (Minolta, Osaka, Japan) equipped with D65 standard illumination and a 10° standard observer angle. The color parameters recorded included L* (lightness; 0 = black, 100 = white), a* (negative values indicating green and positive values indicating red) and b* (negative values indicating blue and positive values indicating yellow) [70]. Before analysis, the instrument was calibrated using a standard white reference tile. Samples were placed on a uniform white background and six measurements per sample were collected at room temperature. The whiteness index (*WI*) and chroma (C*) were also calculated according to Equations (1) and (2), respectively.

(1) $$WI=100-\sqrt{{\left(100-L^{*}\right)}^{2}+{a^{*}}^{2}+{b^{*}}^{2}}$$

(2) $$C^{*}=\sqrt{{a^{*}}^{2}+{b^{*}}^{2}}$$

Six independent measurements were taken for each formulation, and each experiment was performed in duplicate. All results are reported as mean values ± standard deviation.

### 4.5. Microstructure Analysis

#### 4.5.1. Optical and Polarized Microscopy

The microstructure of the different ink formulations was analyzed using an optical microscope (Olympus BX43, Olympus Optical Co., Ltd., Tokyo, Japan) coupled with a digital microscope camera (Basler USB3 Vision, Ahrensburg, Germany). Polarized light microscopy was additionally employed to identify crystalline structures associated with the presence of BW. For sample preparation, a droplet of hot, molten OG, HG or BG was deposited onto a preheated microscope slide and covered with a warmed glass coverslip to maintain the sample in a liquefied state. The prepared inks were then subjected to the previously described storage and equilibration conditions before observation. Micrographs were acquired at 10× magnification and representative images for each formulation were captured using Basler Microscopy Software (v. 2.1; Basler, Ahrensburg, Germany).

#### 4.5.2. Confocal Laser Scanning Microscopy

The microstructure of the ink formulations was further investigated using confocal laser scanning microscopy (CLSM) with a Leica TCS SP5 II system coupled to a Leica DM 6000B inverted microscope (Leica Microsystems, Wetzlar, Germany) operating in fluorescence mode. During ink preparation, Nile Red and Nile Blue were added to the formulations at concentrations of 0.01% *w*/*v* to selectively stain the lipid and aqueous phases, respectively, and the mixtures were thoroughly homogenized to ensure uniform dye distribution. Approximately 2 g of each ink formulation was placed into a Willco- Glass bottom dish (0.17 mm thickness) (WillCo Wells BV, Amsterdam, The Netherlands). Fluorescence excitation was performed using a 488 nm argon laser for Nile Red and a 633 nm HeNe laser for Nile Blue. Images were captured using a 63× oil-immersion objective (numerical aperture = 1.40), with images acquired at depths of 20–30 μm below the coverslip to minimize surface and hydrodynamic artifacts. Micrographs were recorded at a resolution of 512 × 512 pixels in the x–y plane, with eight consecutive scans averaged to enhance image quality. The identification of each ink structure was conducted at 27 °C.

### 4.6. Forward Extrusion Analysis

Forward extrusion testing was employed to evaluate the flow behavior of the inks through a restricted orifice prior to the 3D printing procedure, providing information regarding the extrusion performance and printability of the produced inks. Forward extrusion properties were measured using a forward extrusion rig (HDP/FE) on a TA. XT Plus Texture Analyzer with a 5 kg load cell. After equilibration at 27 °C for 2 h, 100 mL of each formulation was transferred into a polycarbonate cylindrical container (100 mm height, 50 mm diameter) fitted with a base disk having a 3 mm orifice. The test conditions were set to a pre-test speed of 1 mm/s, a test speed of 1 mm/s and a target displacement of 10 mm. Force-displacement curves were recorded throughout the compression process. The maximum force was defined as the extruded hardness, while the mean force value was used to characterize the firmness of the inks. The area under each force–displacement curve was calculated and reported as the work of extrusion (N × mm). The fluctuations observed in the force–displacement profile have been ascribed to the release of entrapped air during extrusion, leading to temporary changes in flow resistance and affecting the stability of the material flow [71]. Three independent measurements were taken for each formulation, and each experiment was performed in duplicate.

### 4.7. Rheological Characterization

The rheological properties of the ink formulations were characterized using an Anton Paar MCR 92 rheometer (Anton Paar GmbH, Graz, Austria) operated in strain-controlled mode and equipped with a Peltier temperature control system. All measurements were performed using a parallel-plate geometry (25 mm diameter) with a fixed gap of 1 mm. Rheological tests were conducted in duplicate at 27 °C. Two independent measurements were taken for each formulation, and each experiment was performed in duplicate.

#### 4.7.1. Apparent Viscosity

Shear viscosity measurements were performed under rotational flow conditions by varying the shear rate from 0.1 to 100 1/s. The shear-thinning behavior of the inks was quantified by fitting the linear region of the shear rate–viscosity curves to the Ostwald-de Waele power law model (Equation (3)):

(3) $$\eta =Κ\gamma^{n-1}$$

where *η*, *γ*, *K*, and *n* represent the apparent viscosity, shear rate, consistency coefficient, and flow behavior index, respectively.

#### 4.7.2. Thixotropy Analysis

The structural recovery behavior of the inks following the application of high deformation was evaluated using a three-interval oscillatory step test conducted at a constant frequency of 1 Hz under strain-controlled conditions [72]. This protocol simulates the viscosity changes experienced by inks during the 3D printing process through three sequential stages [59]: (i) a low-deformation interval at 1 1/s representing the material at rest, (ii) a high-deformation interval at 100 1/s, corresponding to structural breakdown during extrusion through the printer nozzle and (iii) a second low-deformation interval at 1 1/s simulating structural recovery after printing. The percentage recovery of the inks, used as an indicator of viscoelastic regeneration, was calculated according to Equation (4):

(4) $$Recovery\;(\%)=\left(\frac{\eta_{\infty }-\eta_{shear}}{\eta_{0}-\eta_{shear}}\right)100$$

where *η*_0_ is the final equilibrium apparent viscosity at the first static stage (1 1/s); *η_shear_* is the final equilibrium apparent viscosity at the high shearing stage (100 1/s); *η*_∞_ is the final equilibrium apparent viscosity at the final static stage (1 1/s) [56].

#### 4.7.3. Amplitude Sweep Test

Oscillatory amplitude sweep tests were conducted over a strain range of 0.10–100% at a constant angular frequency of 10 rad/s to determine the linear viscoelastic region (LVR) for subsequent frequency sweep measurements and to evaluate the yield point (*τ_y_*) and flow point (*τ_f_*) of the different ink formulations. The yield point (*τ_y_*) was determined as the stress corresponding to a 10% deviation of *G*′ from its plateau value within the LVR, indicating the beginning of structural breakdown. The flow point (*τ_f_)* was defined as the strain at which the elastic modulus (*G*′) crossed with the viscous modulus (*G*″) [7].

#### 4.7.4. Frequency Sweep Test

Oscillatory frequency sweep measurements were conducted at 27 °C using a parallel-plate geometry with a 1 mm gap under a constant strain of 0.10%, previously determined to be within the LVR. Dynamic rheological tests were performed over an angular frequency range of 1–100 rad/s to evaluate the elastic modulus (*G*′), viscous modulus (*G*″) and the dependence of the complex modulus (G*) on angular frequency (*ω*). Also, the loss tangent (*tanδ*) was calculated to evaluate the viscoelastic behavior (Equation (5)) and a Weak gel model was applied to describe the relationship between complex modulus (G*) and angle frequency (*ω*) as (Equation (6)), as reported in the literature [52]:

(5) $$tan\delta =\frac{G^{\prime \prime }}{G^{\prime }}$$

G* = (*G′^2^* + *G″^2^*)*^0.5^* = *A⋅ω^1/z^* (6)

where *z* represents the interaction number of the inks and *A* denotes the strength of these interactions.

### 4.8. Thermal Behavior

The thermal properties of the inks were analyzed using a microcalorimeter (microCalvet μDSC 7 Evo-1A, Setaram, Caluire-et-Cuire, France) equipped with two parallel 1 mL sample cells. Approximately 200 mg (± 2 mg) of each sample was sealed in one capsule, while the reference capsule contained distilled water. Plain HG was subjected to a heating cycle from 0 to 115 °C, whereas the OG was heated from 30 to 100 °C. For the BG formulations (BG10–BG50), a heating scan from 0 to 115 °C was conducted. All measurements were performed under a continuous nitrogen purge (0.8 bar) at a constant heating rate of 1 °C/min. The instrument was calibrated using pure naphthalene as a standard. Thermal transition parameters, including melting temperature (*T_m_*), crystallization temperature (*T_c_*) and apparent melting/crystallization enthalpy (Δ*H*), were determined from the endothermic and exothermic peaks of the thermograms using CALISTO software v.2.14 software (Setaram, Caluire-et-Cuire, France). All analyses were carried out 24 h after each sample preparation to ensure complete structural stabilization.

### 4.9. Statistical Analysis

The collected data are presented as mean values ± standard deviation (SD). Two independent experimental runs were performed and within each experimental run all measurements were carried out in duplicate, unless otherwise stated. In the case of forward extrusion analysis, three replicate measurements were performed per experimental run. Data were evaluated using one-way ANOVA with a general linear model, with the significance level set at α = 0.05. Tukey’s post hoc test was employed to identify differences among treatments. Statistical analysis was conducted using MINITAB v.16 software (Minitab, Inc., State College, PA, USA).

## Figures and Tables

**Table 1:** Color parameters (L*, a*, b*), whiteness index (*WI*), and chroma (C*) of hydrogel (HG), oleogel (OG) and bigel (BG) ink formulations with different OG:HG ratios (10–50%), denoted as BG10–BG50.

Inks	Color Parameters				
	L*	a*	b*	*WI*	C*
**HG**	65.49 ± 1.56 ^d^	−0.32 ± 0.03 ^a^	7.70 ± 0.22 ^a^	64.63 ± 1.36 ^c^	7.70 ± 0.20 ^a^
**BG10**	88.79 ± 0.79 ^ab^	−0.98 ± 0.04 ^b^	2.95 ± 0.17 ^c^	88.36 ± 0.66 ^a^	2.95 ± 0.16 ^c^
**BG20**	89.61 ± 0.97 ^a^	−1.45 ± 0.15 ^c^	4.21 ± 0.61 ^bc^	88.66 ± 0.68 ^a^	4.21 ± 0.56 ^bc^
**BG30**	86.00 ± 0.48 ^b^	−1.37 ± 0.03 ^c^	4.24 ± 0.30 ^bc^	85.31 ± 0.35 ^b^	4.24 ± 0.28 ^bc^
**BG40**	85.37 ± 2.05 ^c^	−2.52 ± 0.10 ^d^	7.61 ± 0.96 ^a^	83.26 ± 1.54 ^b^	7.61 ± 0.88 ^a^
**BG50**	59.02 ± 1.01 ^e^	−2.94 ± 0.26 ^e^	3.91 ± 1.15 ^bc^	58.71 ± 0.88 ^d^	3.91 ± 1.05 ^bc^
**OG**	61.49 ± 3.05 ^e^	−3.27 ± 0.24 ^f^	4.77 ± 1.00 ^b^	61.04 ± 2.64 ^d^	4.77 ± 0.92 ^b^

Values are expressed as mean ± standard deviation. Different superscript letters within the same column indicate statistically significant differences among inks (*p* < 0.05).

**Table 2:** Forward extrusion properties of hydrogel (HG), oleogel (OG) and bigel (BG) ink formulations with different OG:HG ratios (10–50%), denoted as BG10–BG50, including peak extrusion force, extrusion force at 5 mm and 10 mm displacement, work of extrusion and firmness.

Inks	Peak Extrusion Force (N)	Extrusion Force at 10 mm (N)	Extrusion Force at 5 mm (N)	Work (N × mm)	Firmness (N)
**HG**	10.91 ± 0.35 ^ab^	10.94 ± 1.27 ^ab^	10.68 ± 1.25 ^ab^	109.02 ± 9.97 ^ab^	10.90 ± 1.00 ^ab^
**BG10**	5.68 ± 0.97 ^e^	5.55 ± 1.00 ^e^	5.47 ± 0.90 ^e^	54.71 ± 9.05 ^e^	5.47 ± 0.91 ^e^
**BG20**	7.27 ± 0.76 ^de^	6.57 ± 1.02 ^de^	6.47 ± 0.79 ^de^	64.54 ± 7.94 ^de^	6.45 ± 0.79 ^de^
**BG30**	8.57 ± 1.75 ^cd^	8.35 ± 2.44 ^cd^	8.22 ± 2.18 ^cd^	82.26 ± 21.07 ^cd^	8.22 ± 2.10 ^cd^
**BG40**	9.00 ± 0.55 ^cd^	9.07 ± 0.42 ^bcd^	9.16 ± 0.46 ^bc^	88.92 ± 4.71 ^bc^	9.08 ± 0.55 ^bc^
**BG50**	12.10 ± 0.51 ^a^	12.61 ± 1.32 ^a^	12.39 ± 1.65 ^a^	124.19 ± 11.33 ^a^	12.42 ± 1.13 ^a^
**OG**	9.50 ± 0.83 ^bc^	9.27 ± 1.10 ^bc^	9.07 ± 0.97 ^bc^	90.83 ± 8.43 ^bc^	9.08 ± 0.84 ^bc^

Values are expressed as mean ± standard deviation. Different superscript letters within the same column indicate statistically significant differences among inks (*p* < 0.05).

**Table 3:** Rheological parameters of hydrogel (HG), oleogel (OG) and bigel (BG) ink formulations with different OG:HG ratios (10–50%), denoted as BG10–BG50, determined using the Ostwald-de Waele power law and Weak gel models. The consistency index (*K*) and flow behavior index (*n*) were obtained from the Ostwald-de Waele power law model, while the structural parameter (*A*) and interaction exponent (*z*) were derived from the Weak gel model.

Inks	Ostwald-de Waele Power Law Model			Weak Gel Model		
	*K* (10^5^ mPa·s*^n^*)	*n*	*R* ^2^	*A* (10^4^ Pa·s^1/*z*^)	*z* (−)	*R* ^2^
**HG**	50.98 ± 8.58 ^b^	0.17 ± 0.02 ^a^	0.984	0.10 ± 0.02 ^d^	6.78 ± 0.41 ^b^	0.995
**BG10**	50.57 ± 15.79 ^b^	0.17 ± 0.02 ^a^	0.990	1.19 ± 0.07 ^d^	6.13 ± 1.43 ^b^	0.994
**BG20**	64.98 ± 9.71 ^b^	0.16 ± 0.02 ^a^	0.991	1.72 ± 0.13 ^d^	5.49 ± 5.49 ^b^	0.995
**BG30**	71.79 ± 9.43 ^b^	0.17 ± 0.02 ^a^	0.998	3.74 ± 0.94 ^d^	5.14 ± 0.58 ^b^	0.999
**BG40**	75.03 ± 7.95 ^b^	0.17 ± 0.02 ^a^	0.996	13.95 ± 2.76 ^c^	5.08 ± 0.41 ^b^	0.999
**BG50**	101.96 ± 10.79 ^a^	0.09 ± 0.05 ^b^	0.993	74.26 ± 9.63 ^b^	5.63 ± 1.07 ^b^	0.997
**OG**	107.24 ± 12.28 ^a^	−0.10 ± 0.03 ^c^	0.994	173.46 ± 4.33 ^a^	9.42 ± 0.26 ^a^	0.997

Values are expressed as mean ± standard deviation, together with the corresponding coefficients of determination (*R*^2^). Different superscript letters within the same column indicate statistically significant differences among inks (*p* < 0.05).

**Table 4:** Composition and coding of bigel (BG) inks prepared with different oleogel-to-hydrogel (OG:HG) ratios. Values represent the percent content of each component (beeswax—BW; monoglycerides—MGs; sunflower oil; gelatin—GEL; guar gum—GG; and water) in the total ink formulation.

Inks	Mixing Ratio		OG Phase			HG Phase		
	OG (%)	HG (%)	BW (%)	MGs (%)	Sunflower Oil (%)	GEL (%)	GG (%)	Water (%)
**HG**	0	100	0	0	0	4.0	1.0	95.0
**BG10**	10	90	0.6	0.4	9	3.6	0.9	85.5
**BG20**	20	80	1.2	0.8	18	3.2	0.8	76.0
**BG30**	30	70	1.8	1.2	27	2.7	0.7	66.5
**BG40**	40	60	2.4	1.6	36	2.4	0.6	57.0
**BG50**	50	50	3.0	2.0	45	2.0	0.1	47.5
**OG**	100	0	6.0	4.0	90	0.0	0.0	0.0

## Data Availability

The data presented in this study are available on request from the corresponding author.

## Abbreviations

The following abbreviations are used in this manuscript:

*A*	Structural parameter
*a**	Red–green color coordinate
*b**	Yellow–blue color coordinate
BG	Bigel
BW	Beeswax
*C**	Chroma
CLSM	Confocal Laser Scanning Microscopy
DSC	Differential Scanning Calorimetry
Δ*H*	Enthalpy change
*G*′	Storage modulus
*G*″	Loss modulus
*G**	Complex modulus
GEL	Gelatin
GG	Guar gum
HG	Hydrogel
*K*	Consistency index
*L**	Lightness
LVR	Linear Viscoelastic Region
MGs	Monoglycerides
*n*	Flow behavior index
OG	Oleogel
OM	Optical Microscopy
PLM	Polarized Light Microscopy
*R*^2^	Coefficient of determination
*tanδ*	Loss tangent
*T**ₘ*	Melting temperature
*T_c_*	Crystallization temperature
*WI*	Whiteness Index
*z*	Interaction exponent (Weak Gel model)
*γ*	Shear rate
*η*	Apparent viscosity
*τ_f_*	Flow point
*τ_y_*	Yield point
*ω*	Angular frequency
3ITT	Three-Interval Thixotropy Test
